# Carbon-bearing silicate melt at deep mantle conditions

**DOI:** 10.1038/s41598-017-00918-x

**Published:** 2017-04-12

**Authors:** Dipta B. Ghosh, Suraj K. Bajgain, Mainak Mookherjee, Bijaya B. Karki

**Affiliations:** 1 School of Electrical Engineering and Computer Science, Department of Geology and Geophysics, Center for Computation and Technology, Louisiana State University, Baton Rouge, LA 70803 USA; 2 Earth, Ocean and Atmospheric Sciences, Florida State University, Tallahassee, FL 32306 USA

## Abstract

Knowledge about the incorporation and role of carbon in silicate magmas is crucial for our understanding of the deep mantle processes. CO_2_ bearing silicate melting and its relevance in the upper mantle regime have been extensively explored. Here we report first-principles molecular dynamics simulations of MgSiO_3_ melt containing carbon in three distinct oxidation states - CO_2_, CO, and C at conditions relevant for the whole mantle. Our results show that at low pressures up to 15 GPa, the carbon dioxide speciation is dominated by molecular form and carbonate ions. At higher pressures, the dominant species are silicon-polyhedral bound carbonates, tetrahedral coordination, and polymerized di-carbonates. Our results also indicate that CO_2_ component remains soluble in the melt at high pressures and the solution is nearly ideal. However, the elemental carbon and CO components show clustering of carbon atoms in the melt at high pressures, hinting towards possible exsolution of carbon from silicate melt at reduced oxygen contents. Although carbon lowers the melt density, the effect is modest at high pressures. Hence, it is likely that silicate melt above and below the mantle transition zone, and atop the core-mantle boundary could efficiently sequester significant amounts of carbon without being gravitationally unstable.

## Introduction

Volatiles such as carbon and hydrogen are important elements that are often present in shallow mantle as CO_2_ and H_2_O fluids, which help in driving volcanic eruptions^1, 2^. In the mantle, carbon also occurs as carbonates^3–5^, and as diamond and metal carbides under reducing mantle conditions^6–8^. Unlike hydrogen, carbon has very low solubility in major mantle silicate minerals^9, 10^. It is likely that most of the carbon gets partitioned into molten silicates^11–15^. Although the present-day mantle is mostly solid, partial melts as evidenced from anomalous geophysical observations exist in various mantle conditions such as the mantle transition zone and ultra-low velocity zones at the core mantle boundary^16–18^. In addition, in the early history of the Earth, most of the silicate mantle might have been molten, resulting in a large scale magma ocean^19^. The remnant of such magma ocean is likely to have been preserved in the lowermost part of the present-day mantle^17–20^. It is possible that silicate magmas could have actually served as one of the largest repositories of mantle volatiles including carbon throughout the earth’s history. However, we know very little about the incorporation of carbon in silicate melts and how it influences the atomistic scale structure and thus physical properties of the melt at pressure-temperature conditions pertaining to the whole mantle.

Extensive studies on the speciation of carbon in silicate melts^21–23^, the solubility of carbon^24, 25^, and the effect of carbon on the melt density^13, 14, 26^ have been mostly confined to conditions relevant for the deep crust and upper mantle. Recent numerical simulations on carbon-bearing basaltic and kimberlitic melts are also confined to pressures up to 15 GPa^27, 28^. Here we investigate the high-pressure behavior of carbon bearing silicate melt using first-principles molecular dynamics (FPMD) simulation method^29^. In order to explore the effects of oxygen content on the speciation of carbon, we consider three distinct scenarios of dissolved carbon in MgSiO_3_ system as carbon dioxide (CO_2_), carbon monoxide (CO), and elemental carbon (C). We also explore a wide range of carbon concentration in terms of 5.2, 16.1, and 30.5 wt.% CO_2_ (and equivalent CO and elemental carbon proportions). The calculated results on the structure and thermodynamic properties of carbon-bearing silicate melts under conditions of the whole mantle allow us for the first time to check whether carbon in its oxidized and reduced forms can be effectively be sequestered in high-pressure silicate melts and whether these carbon-bearing melts are sufficiently dense with respect to the surrounding solid mantle to be gravitationally stable.

**Figure 1:**
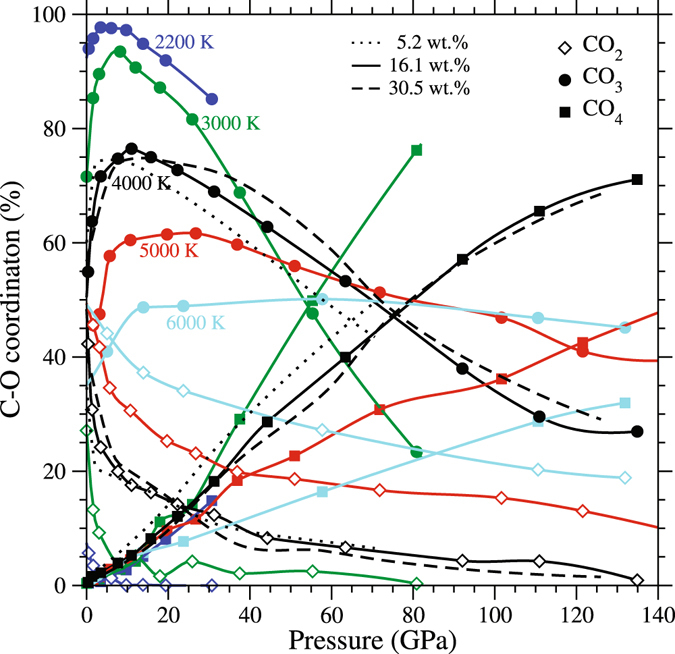
Speciation of carbon dioxide in MgSiO_3_ liquid. Calculated abundances of different C-O coordination species (correspondingly, CO_2_ speciation forms) in MgSiO_3_ liquid: CO_2_ molecules (diamonds), carbonate ions (circles), and CO_4_ groups (squares). The results for 16.1 wt.% CO_2_ are shown as a function of pressure at various temperatures (lines with symbols) compared with other concentrations at 4000 K.

**Figure 2:**
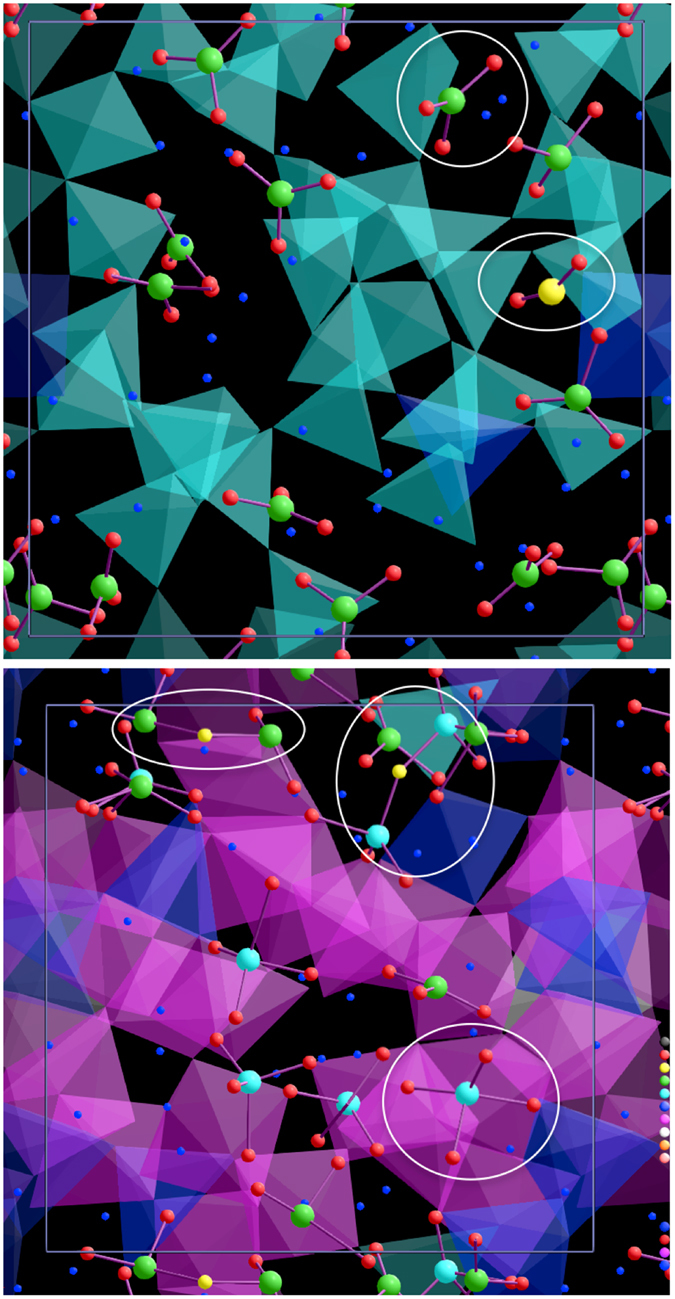
Visualization snapshots of the melt structure. Various CO_n_ species (marked with ellipses) and the Si-O tetrahedra (cyan), pentahedra (blue), and octahedra (pink) are present in the melt at the zero pressure and 2200 K (*top*), and 92 GPa and 4000 K (*bottom*) for 16.1 wt.% concentration. C_2_O_5_ and C_2_O_7_ can be seen in the compressed melt. Tiny blue spheres represent Mg atoms in both snapshots.

We characterize the speciation of carbon dioxide in MgSiO_3_ liquid in terms of coordination environments of carbon and oxygen (Fig. 1). The C-O coordination mainly consists of two-fold species (a molecular CO_2_) and three-fold species (a carbonate ion, ${{\rm{CO}}}_{3}^{2-}$). Their respective abundances at zero pressure and 2200 K are 5.7 and 94%. Both molecular and carbonate species have been experimentally detected in silicate melts and glasses below 10 GPa^21–23^. At higher pressures, the speciation changes: At the expense of CO_2_ molecules, the abundance of carbonate ions increases, reaching as high as 98% at 5 GPa and 2200 K. On further compression, the ${{\rm{C}}{\rm{O}}}_{3}^{2-}$ population declines and is replaced by tetrahedrally coordinated species (CO_4_) also found in solid carbonate minerals^4, 5^. The CO_4_ species becomes dominant at pressures above 60 GPa. Strong C-O short-range order is manifested in the corresponding radial distribution function (RDF) with a well-defined peak (Supplementary Fig. S1). The CO_2_ molecules and carbonate ions exist mostly in isolation at low pressures (Fig. 2, top) because oxygen rarely is coordinated with two carbons. However, our simulations for highly compressed system indicate the presence of polymerized di-carbonate complexes, C_2_O_n_ (Fig. 2, bottom). Such di-carbonates have been also predicted previously in basaltic and kimberlitic melts^28^. The polymerized complexes are known to form when carbon dioxide dissolves in a carbonate melt via^30^ CO_2_ + ${{\rm{CO}}}_{3}^{2-}$ ↔ ${{\rm{C}}}_{2}{{\rm{O}}}_{5}^{2-}$. Simulations show other types of species including C_2_O_6_ and tetrahedrally coordinated di-carbonates, C_2_O_7_. The presence of –C-C- dimers at high pressure is manifested in the C-C RDF with new peak appearance at ~1.5 Å (Supplementary Fig. S1).

The Si-O RDF and mean coordination remain largely unaffected by the carbon (Supplementary Figs S1 and S2). However, the Mg-O RDF and mean coordination are somewhat enhanced as a function of carbon-dioxide content in the silicate melt. Our results show that the conversion of molecular CO_2_ → ${{\rm{CO}}}_{3}^{2-}$ → CO_4_→ polymerized di-carbonates upon compression involves increased sharing of oxygen with both magnesium and silicon. Almost all CO_2_ molecules and majority carbonate ions (about 65% at 2200 K and 0 GPa) exist as free units at low pressure, i.e., these species are majorly bound to magnesium via Mg-O-C connections (Fig. 2, top). This would imply that as the mantle melt upwells through the upper mantle, the dominant ${{\rm{CO}}}_{3}^{2-}$ species might serve as nucleating seeds for the formation of MgCO_3_ from a carbonated silicate melt. Upon segregation of volatiles in the form of MgCO_3_, the remaining melt composed largely of networked SiO_2_ units would freeze. Hence, faster removal of carbonated species and precipitation as MgCO_3_ may account for elevating the solidus temperature to a volatile free silicate melting of >2000 K compared to that of 600 K for magnesite. At high pressure, carbon dioxide is much more integrated in the melt with mean C-O coordination exceeding 3.5 (Supplementary Fig. S2). Pressure enhances the formation of Si-O-C connections as shown by the presence of the Si-C RDF peak (Supplementary Fig. S1) and over 90% carbons form such polyhedral connections at pressures above 50 GPa. Upon compression, the nature of bonding in carbon species appears to evolve from ionic to covalent. The polyhedral association occurs mostly via the formation of bonds between carbon and non-bridging oxygen. Besides, there exist bridging CO_n_ groups at elevated pressures arising from the formation of bonds between carbons and bridging oxygens.

**Figure 3:**
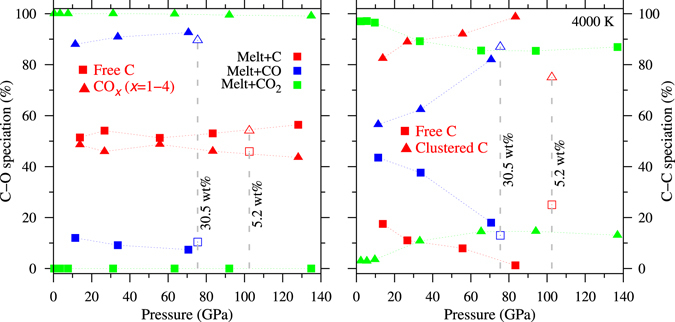
Speciation of CO and C components in MgSiO_3_ liquid. Abundances of C-O and C-C speciation as a function of pressure at 4000 K for three cases of carbon incorporation in the melt: a) oxidized form of CO_2_, b) intermediate form of CO, and c) reduced form of elemental carbon. The amount of carbon in each case is equivalent to that of 16.1 wt.% CO_2_. The C-O speciation is divided into two groups: free C atoms (not coordinated/bonded with any oxygen), and the other C atoms which are bonded to one or more oxygen atoms. Similarly, the C-C speciation is divided into two groups: free C atoms (not coordinated/bonded with any other carbon), and the other C atoms which are bonded/coordinated with one or more carbon atoms. Also shown are the results for 5.2 and 30.5 wt.% equivalent cases at high pressures.

Our results on elemental carbon (C) and carbon monoxide (CO) in MgSiO_3_ liquid help us understand how the carbon speciation in silicate melt is affected under oxygen-poor, i.e., reducing conditions. We find that carbon incorporation occurs via both C-O and C-C bonding. In reduced silicate melts, the C-O coordination is severely suppressed whereas the C-C coordination is enhanced relative to the more oxidized CO_2_ bearing silicate melt (Fig. 3). For the most reduced condition with elemental carbon in the amount corresponding to 16.1 wt.% CO_2_, about 50% carbons are bonded with oxygen. More than 80% carbon atoms are coordinated with each other forming carbon-carbon clusters, that is, the C-C bonds prevail in the melt. This clustering is less prominent at lower carbon content but it is enhanced with pressure. In contrast, 90% or more C atoms are not connected with each other in the oxidized case at pressures below 20 GPa so the presence of polymerized species such as di-carbonates is severely suppressed. The CO component shows an intermediate level of C-C bonding and clustering. The high C-C bonding activity of elemental carbon-bearing MgSiO_3_ was previously predicted in the C-O-H-Fe system under conditions of reduced activity of oxygen^31^. Hence, our results demonstrate that under reducing conditions expected in the present day lower mantle, the carbon atoms may form clusters in silicate melt or may tend to exsolve out from the silicate melt and possibly form separate metal carbide melt at the core-mantle boundary conditions as recently suggested^32^.

**Figure 4:**
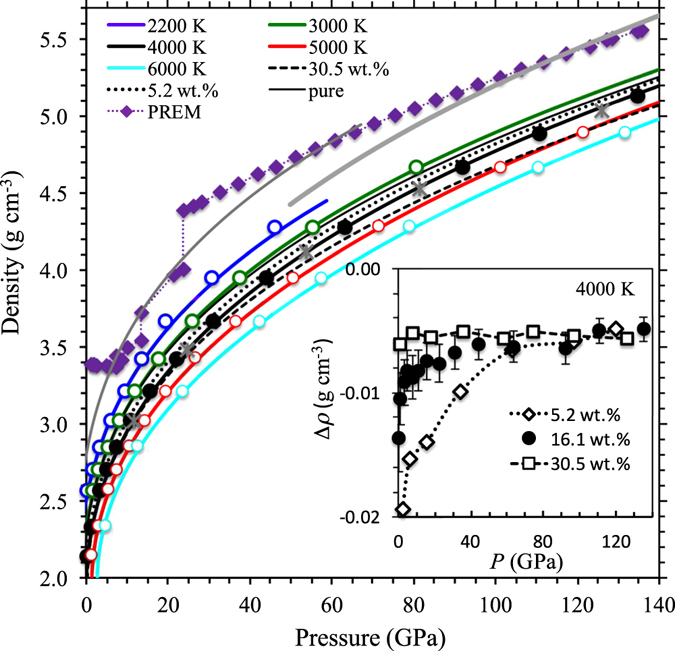
Density-pressure profiles of MgSiO_3_ liquid. The calculated density results for the melt containing 16.1 wt.% CO_2_ at various temperatures (thick lines with circles), compared with the results for liquids containing 5.2 and 30.5 wt.% CO_2_ (black dotted and dashed lines) and the pure liquid (black thin line) at 4000 K. In all cases, the lines represent the equation of state fits. Also shown is the liquid density at 4000 K (asterisks) for the same amount of elemental carbon as in case of 16.1 wt.% CO_2_. The estimated density profiles of (Mg_1−*x*_,Fe_*x*_)SiO_3_ melt with 5 wt.% CO_2_ at 2200 K (with *x* = 0.22) and 4000 K (with *x* = 0.27) shown by thin and thick gray curves are compared with the seismic density profile (PREM)^38^. The inset shows the changes in the silicate melt density due to the CO_2_ component at 4000 K for three concentrations calculated as *∆ρ* = (*ρ*_mix_ − *ρ*_pure_)/*c*. Here, *ρ*_mix_ and *ρ*_pure_ are the densities of the carbonated and pure MgSiO_3_ liquids, respectively, and *c* is the wt.% of CO_2_ content.

The equation of state of the carbon bearing MgSiO_3_ liquid as in the case of pure liquid^33^ can be accurately described with *P*(*ρ*, *T*) = *P*(*ρ*, *T*_0_) + *B*_TH_(*ρ*)(*T* − *T*_0_), where *P*(*ρ*, *T*) is the total pressure at temperature *T* and density *ρ*. The reference isotherm *P*(*ρ*, *T*_0_) at *T*_0_ = 3000 K is the third order Birch-Murnaghan equation for each composition, with the fit parameters being somewhat sensitive to CO_2_ concentration. The derived equation of state agrees well with experimental results^13, 14^ (Table 1). The thermal pressure coefficient *B*_TH_ defined as (d*P*/d*T*)_V_ increases by a factor of ten over the compression range explored in this study and is largely insensitive to composition (Supplementary Fig. S3). All cases of carbon-bearing MgSiO_3_ liquids are highly compressible but always remain less dense than the pure (carbon free) liquid (Fig. 4). In the low-pressure regime, the CO_2_ component decreases the melt density by large amounts (Fig. 4, Inset). However, the density contrasts between the carbon bearing and pure melts decreases considerably at higher pressures. The density effects of carbon in the reduced forms of C and CO are similar to that of CO_2_ for the same amount of carbon (Fig. 4, Supplementary Fig. S4).

**Table 1:** Equation of state parameters.

	Pure MgSiO_3_ melt	5.2 wt.% CO_2_	16.1 wt.% CO_2_	30.5 wt.% CO_2_	Expt.^13^ basalt	Expt.^14^ peridotite
*ρ*_0_ (g cm^−3^)	2.56 ± 0.02	2.43 ± 0.03	2.33 ± 0.03	2.31 ± 0.04	—	—
*K*_0_ (GPa)	17.9 ± 1.2	15.7 ± 1.5	14.4 ± 1.5	12.9 ± 1.8	16.0 ± 1.0	22.9 ± 1.4
*K*´_0_	6.9 ± 0.2	6.5 ± 0.2	6.3 ± 0.3	7.1 ± 0.3	5.2 ± 0.2	7.4 ± 1.4

The 3^rd^ order Birch Murnaghan equation of state *P*(*ρ*, *T*_0_) used to represent the reference isotherm of *T*_0_ = 3000 K. The experimentally adopted parameters for carbonated basaltic melt (containing 5 wt.% CO_2_)^13^ using the data at 16–20 GPa and 2573 K and those for carbonated peridotitic melt (containing 2.5 wt.% CO_2_)^14^ using data up to 3.8 GPa at 2100 K.

Note: The calculated values of linear coefficient (*B*_TH_) in the thermal pressure term as a function of compression for pure and 5.2. 16.1, and 30.5 wt.% CO_2_-bearing silicate liquids can be accurately described by the same equation: *B*_TH_(*ρ*) = 17.47 − 17.64*ρ* + 5.81*ρ* ^2^ − 0.51*ρ* ^3^, where *ρ* is the melt density.

The calculated density reductions (Δ*ρ*) of 0.015 to 0.005 g cm^−3^ per wt.% CO_2_ between 10 and 140 GPa are smaller than those due to the water component (∼0.03 g cm^−3^ per wt.% H_2_O^34^). Carbon-induced partial melting has been proposed atop mantle transition zone and in the uppermost parts of the lower mantle either owing to the changes in the oxidation state^6, 7, 15^ and/or due to the lowering of solidus temperatures (in the presence of minor amounts of alkalies, for instance) so that the solidus intersects the mantle adiabat^35, 36^. Based on the carbon-associated density differences found by this study, carbon-bearing silicate melts provide us with a viable mechanism to explain geophysical anomalies of the deep mantle^16, 18^. Considering a peridotitic melt density of 3.66 gcm^−3^ at 13.6 GPa and 1873 K^37^ and the mantle density of 3.54 gcm^−3^ at 410 km depth^38^, we find that with Δρ = 0.015 gcm^−3^ per wt.% CO_2_, the addition of up to 8 wt.% of CO_2_ can produce a neutrally buoyant silicate melt at the top of the mantle transition zone. To assess the buoyancy situation at greater depths, we estimate the density of carbon-bearing MgSiO_3_ liquid by including iron contribution (Supplementary text S1) for which we use the iron partitioning coefficient of ≤0.4 between the bulk solid mantle and partial melt^39^, and the bulk iron content of of 0.1. The estimated density of the silicate melt containing 5 wt.% CO_2_ at 23.5 GPa and 2200 K (Fig. 4) is greater than the mantle density of 4.0 gcm^−3^ at 660 km depth^38^. Similarly, the melt density at 4000 K exceeds the mantle density near the CMB (Fig. 4).

**Figure 5:**
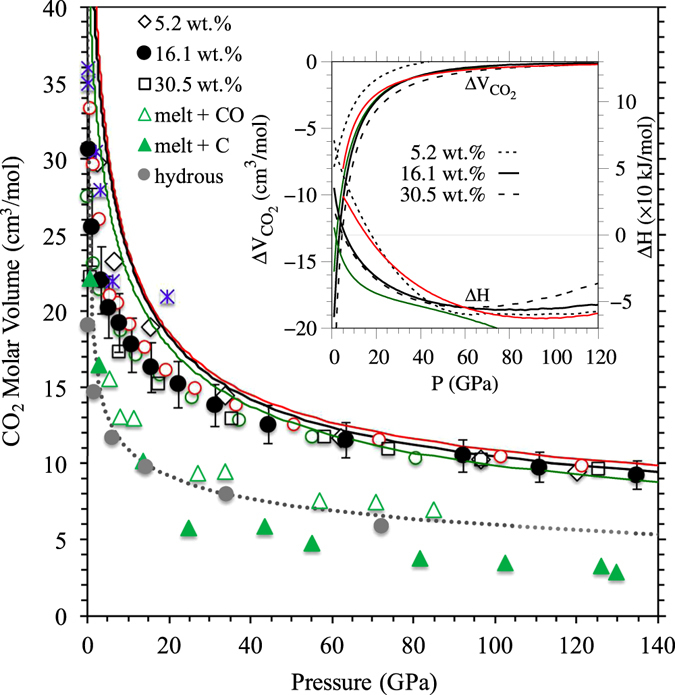
Mixing properties of melt-CO_2_ solution. The calculated partial molar volume (${\bar{V}}_{{{\rm{CO}}}_{2}}$) of the carbon dioxide component in MgSiO_3_ liquid at 3000 K (green circles), 4000 K (black circles), and 5000 K (red circles) for 16.1 wt.% CO_2_ and at 4000 K also for 5.2 and 30.5 wt.%. The partial molar volume is defined as ${\bar{V}}_{{{\rm{CO}}}_{2}}$ = (*V*_mix_ − *V*_pure_)/*n*, where *V*_mix_ and *V*_pure_ are the volumes of the carbonated and pure silicate liquids, respectively, and *n* is the number of CO_2_ molecules added to the system. The calculated molar volume of pure CO_2_ fluid is shown along the corresponding isotherms (curves). The experimental data (asterisks) are for basalt at 19.5 GPa and 2573 K^13^, peridotite at pressures up to 3.8 GPa and 2050 K^14^, and komatite at 5.5 GPa and 2123 K^26^, containing 2.3 to 5.5 wt.% CO_2_. Also calculated are the partial molar volumes of the CO (open triangles) and C (filled triangles) components in the melt at 3000 K for the amount of carbon corresponding to 16.1 wt.% CO_2_. The partial molar volume of H_2_O in the hydrous melt (filled grey circles) from previous calculations and the molar volume of pure water (dotted curve) at 3000 K are shown for comparison^34, 42^. The inset shows the calculated volume of mixing: ∆*V* = ${\bar{V}}_{{{\rm{CO}}}_{2}}$ − ${V}_{{{\rm{CO}}}_{2}}$ and enthalpy of mixing:Δ*H*=${\bar{H}}_{{{\rm{CO}}}_{2}}$ − ${H}_{{{\rm{CO}}}_{2}}$ at the corresponding conditions. Here, ${\bar{H}}_{{{\rm{CO}}}_{2}}$ = (*H*_mix_ − *H*_pure_) is the enthalpy per CO_2_ formula unit in the melt and ${H}_{{{\rm{CO}}}_{2}}$ is that of pure CO_2_ fluid.

The calculated thermodynamic results allow us to explore the behavior of the silicate melt-CO_2_ solution. The calculated value of partial molar volume ${\bar{V}}_{{{\rm{CO}}}_{2}}$ for the melt containing 5.2 wt.% CO_2_ is 33 ± 4 cm^3^/mol at zero pressure and 3000 K. As pressure increases, ${\bar{V}}_{{{\rm{CO}}}_{2}}$decreases rapidly initially and then gradually (Fig. 5). The partial molar volume ${\bar{V}}_{{{\rm{CO}}}_{2}}$ is in good agreement with the experimental estimates for basaltic^13^, peridotitic^14^, and komatitic^26^ melts at low pressures. Pressure systematically suppresses both compositional and thermal effects so the values of ${\bar{V}}_{{{\rm{CO}}}_{2}}$ for all cases converge to 10 cm^3^/mol above 100 GPa. Compared to the partial molar volume of the CO_2_ component, the estimated partial molar volumes of the CO and C components in MgSiO_3_ liquid are systematically smaller, converging to 7 and 3.5 cm^3^/mol, respectively, at high pressure (Fig. 5). We have also calculated the the molar volume (${V}_{{{\rm{CO}}}_{2}}$) of pure carbon dioxide fluid as a function of pressure. Our results show that the partial molar volume of melt CO_2_ is significantly smaller than the molar volume (${V}_{{{\rm{CO}}}_{2}}$) of pure fluid at zero pressure. Two volumes approach each other rapidly as pressure increases (Fig. 5). The volume of the melt-CO_2_ solution, Δ*V* = ${\bar{V}}_{{{\rm{CO}}}_{2}}$ − ${V}_{{{\rm{CO}}}_{2}}$, is large and negative at low pressure (Fig. 5, Inset). For all isotherms and concentrations, its magnitude decreases rapidly with pressure and becomes nearly zero, implying an ideal solution within the computational uncertainty. Predicted negative Δ*V* (=d(Δ*G*_mix_)/d*P* < 0) means that the Gibbs free energy of mixing decreases with pressure. The calculated Δ*H* indeed decreases from positive values at low pressure to negative values at high pressure (Fig. 5, Inset) for all temperatures and concentrations. The melt-CO_2_ solution is thus energetically favorable over a wide pressure range for carbon contents.

Our study based on first-principles molecular dynamics simulations provides valuable insights into the energetics of CO_2_ miscibility in the silicate melts. The CO_2_ solubility remains high over most of the mantle pressure regime. However, under reducing conditions, our results indicate carbon-carbon clusters implying that carbon solubility in silicate melts may reach a limit. It is generally known that the oxygen fugacity decreases significantly with increasing depth and the lower mantle is likely to be strongly reducing^8, 40^. The oxidation state in deeper parts probably is inhomogeneous, consisting of locally or regionally oxidized domains associated with deeply subducting slabs where CO_2_-bearing silicate melts could be stabilized^15, 36^. An upwelling silicate mantle could undergo redox melting across the lower mantle and mantle transition zone discontinuities (i.e., 660 km depth) so the mantle melting itself could be oxidizing^7, 8^.

Although subducted carbonate-rich oceanic crust could transfer carbon up to a depth of 700 km, deeper subduction of slabs containing reduced carbon as metal carbide and subsequent melting to form carbon-bearing metallic melts has been recently proposed^32, 36^. This carbon-rich melt is also likely to replenish the carbon in the deep mantle and eventually interact with any deep-seated silicate melt. If the present day carbon budget of the mantle has its origin dating back to the early Earth core formation period when a metallic-core segregated from a silicate mantle, the process might have sequestered significant amounts of carbon in a residual dense basal magma ocean at the bottom of the mantle and in possibly melt pockets associated with local anomalies detected in other parts of the mantle. Irrespective of the origin, such carbon may remain dissolved in silicate melt near the CMB perhaps in the oxidized forms because more of disproportionated metallic iron from the melt could have descended into the core thereby implying increased oxidation level. However, if the deep mantle is indeed strongly reduced as generally assumed, the reduced forms of carbon may still prefer the silicate melt (perhaps being incorporated as carbon-carbon clusters and/or metal carbides^32^) over the solid mantle. Once incorporated, the carbon in silicate melts is likely to be sequestered owing to greater thermodynamic and gravitational stability. That mantle contained in deep-seated dense melts and perhaps in early magma ocean should represent an important part of the global carbon cycle^11^.

## Methods

First principles molecular dynamics simulations within the local density approximation and projector augmented wave potentials were performed using the VASP program^29^. We explored three distinct scenarios: (a) MgSiO_3_ melts with dissolved carbon dioxide (with 5.2, 16.1, and 30.5 wt.% CO_2_), (b) MgSiO_3_ with dissolved CO, and (c) MgSiO_3_ with dissolved elemental carbon (C). The supercell contained 32 formula units of MgSiO_3_ for the pure system, 32 formula units of MgSiO_3_ and 4 formula units of CO_2_ for 5.2 wt.% CO_2_, 32 formula units of MgSiO_3_ and 14 formula units of CO_2_ for 16.1 wt.% CO_2_, and 16 formula units of MgSiO_3_ and 16 formula units of CO_2_ for 30.5 wt.% CO_2_. The pure CO_2_ system used 72 atoms. For silicate melts with dissolved C and CO, the same numbers of corresponding formula units (C or CO) as in the case of CO_2_ were included in the selected simulations.

Many canonical *NVT* ensembles were generated to cover a volume range of *V* = 1.5*V*_X_ to 0.5*V*_X_ (where *V*_X_ = 2067.1 Å^3^) corresponding to the entire mantle pressure regime (0 to 140 GPa), and a temperature range of 2200 to 6000 K. A cutoff energy of 400 eV (with Gamma point Brillouin zone sampling) was used, requiring the Pulay stress correction of 3 to 8 GPa for the volume range considered. At each volume, the system was melted and thermalized at 8000 K, and then subsequently quenched down to desired lower temperatures. The run durations with a time step of 1 femtosecond ranged from 50 to 300 picoseconds, depending on *V*-*T* conditions. Unlike most previous simulations of liquids, no empirical corrections were applied. The liquid state at each condition was confirmed by examining the radial distribution functions (RDF’s) and mean square displacements (MSD’s). The radial distribution function for each atomic pair species show a well-defined first peak in most cases and even a second peak sometimes, then converges to the unity value implying strong short-range order but no long-range order (Supplementary Fig. S1). The MSD plots show that all atomic species reached diffusive regime within the simulation durations (Supplementary Fig. S5). The calculated results on pressure, energies and various structural parameters were well converged with respect to the system size and runtime. Further details can be found in previous publications^41, 42^.

## Electronic supplementary material

Computational details
