# Supplementary material for: Carbon-bearing silicate melt at deep mantle conditions
Source: Sci Rep. 2017 Apr 12;7:848. doi: 10.1038/s41598-017-00918-x (PMC5429813; doi:10.1038/s41598-017-00918-x)
Supplement: Supplementary file 1 — Computational details [file 41598_2017_918_MOESM1_ESM.pdf]

Supplementary Information

Carbon-bearing Silicate Melt at Deep Mantle Conditions

Dipta B. Ghosh, Suraj K. Bajgain, Mainak Mookherjee & Bijaya B. Karki

Supplementary figure S1. Radial distribution function matrix (symmetric) plot for a 16.1 wt.% CO_2-bearing $MgSiO_3$ liquid at 0.5 GPa and 2200 K (red, blue, and black curves), and 92 GPa and 4000 K (green curves). In each plot, the vertical axis represents the radial distribution function value and the horizontal axis represents the distance in Å. There are four like atom pairs (diagonal plots) and six unlike atom pairs (off-diagonal plots: the lower plots use the global scale whereas the upper plots are in the local scale). The two vertical lines are used to mark the first peak (red line) and the minimum after the first peak (blue line).

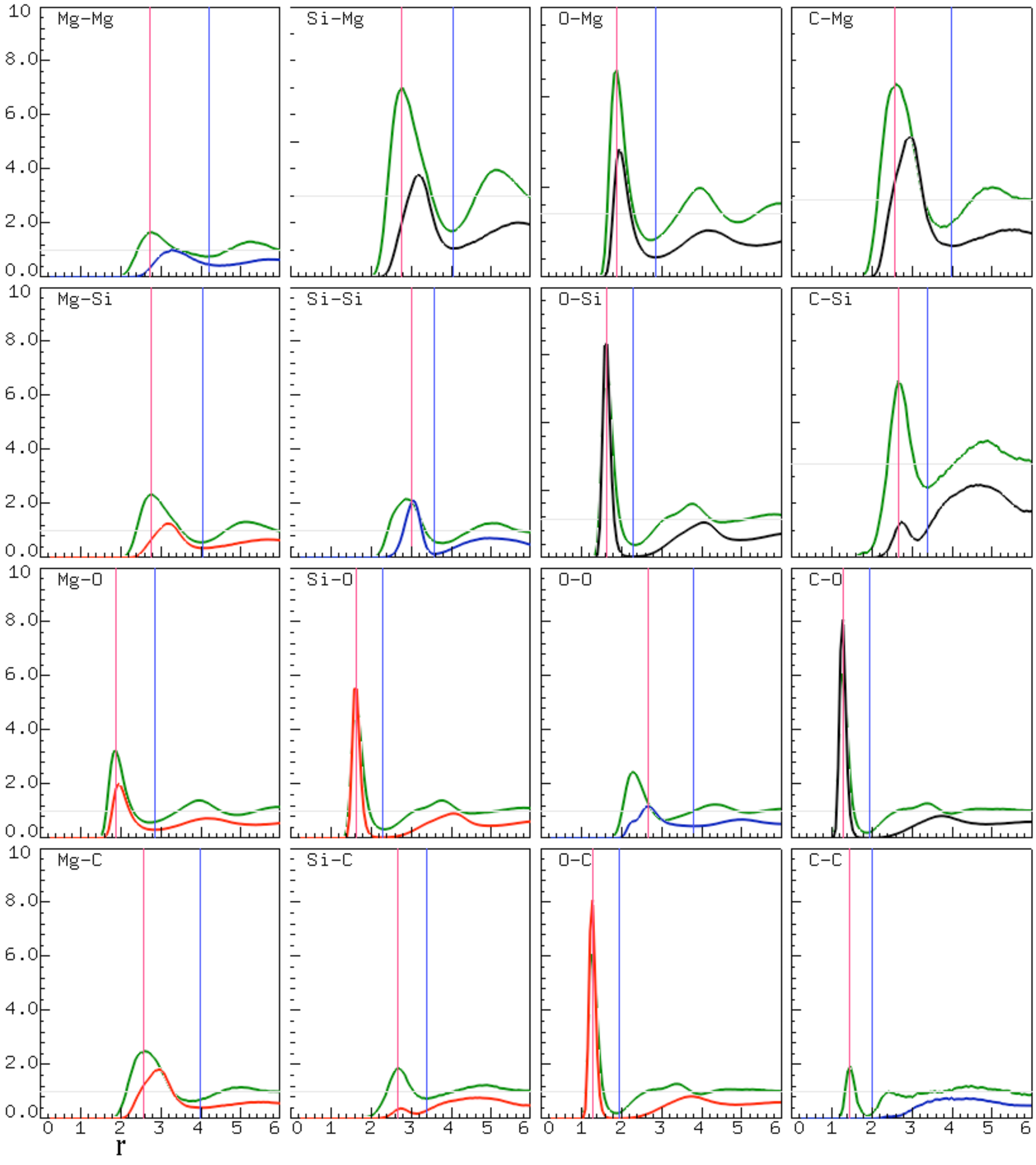

Supplementary figure S2. Calculated mean C-O, Mg-O and Si-O coordination numbers of three carbonated $MgSiO_3$ liquids (with 5.2, 16.1 and 30.5 wt.% CO_2) plotted as a function of pressure at different temperatures. Also shown are the Mg-O and Si-O coordination numbers for the pure silicate liquid (grey filled symbols). The differences in the C-O coordination between different carbon concentrations are small. However, the Mg-O and Si-O coordination numbers of a carbonated silicate liquid tend to be larger compared to the pure liquid numbers and also with increasing CO_2 concentration, particularly at higher pressures. This means that the Mg and Si atoms also form bond with oxygen atoms from the added carbon dioxide component.

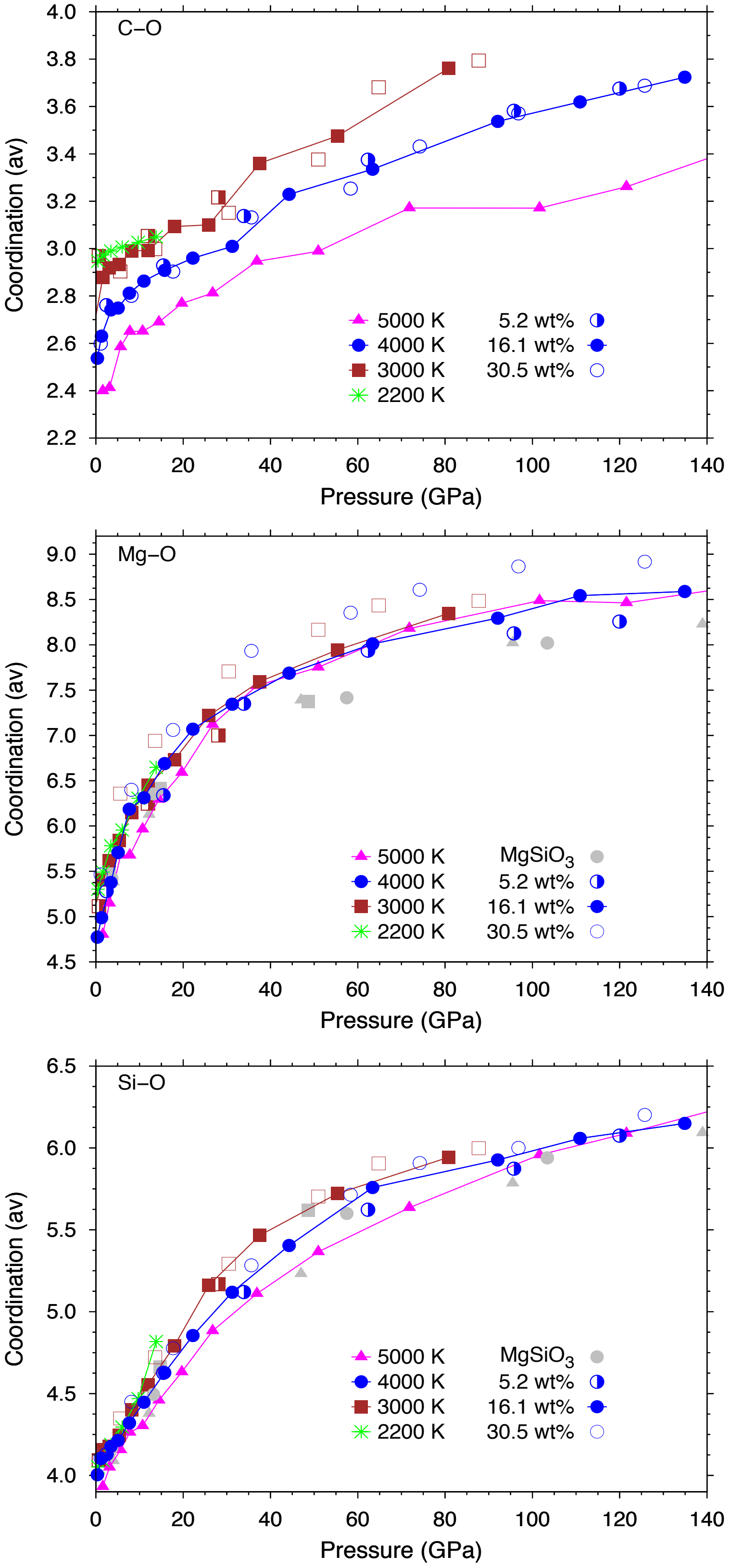

Supplementary figure S3. Variation of the thermal pressure coefficient B_{TH} with density (ρ) for CO_2-bearing and pure $MgSiO_3$ liquids. Note that the thermal pressure is linear in temperature (T) as defined by $P_{TH} = B_{TH}(T-T_0)$, where T_0 represents the reference isotherm. The same cubic equation, $B_{TH}(\rho) = 17.47 - 17.64\rho + 5.81\rho^2 - 0.51\rho^3$, can accurately describe the calculated linear coefficient values as a function of compression for all compositions: 0, 5.2. 16.1, and 30.5 wt.% CO_2-bearing silicate liquids. The equation of state curves at 4000 K shown in Fig. 2 for all compositions were obtained by using their respective reference (3rd-order Birch Murnaghan) equation of state, $P(\rho,T_0)$ given in Table 1, and the same $B_{TH}(\rho)$ shown here.

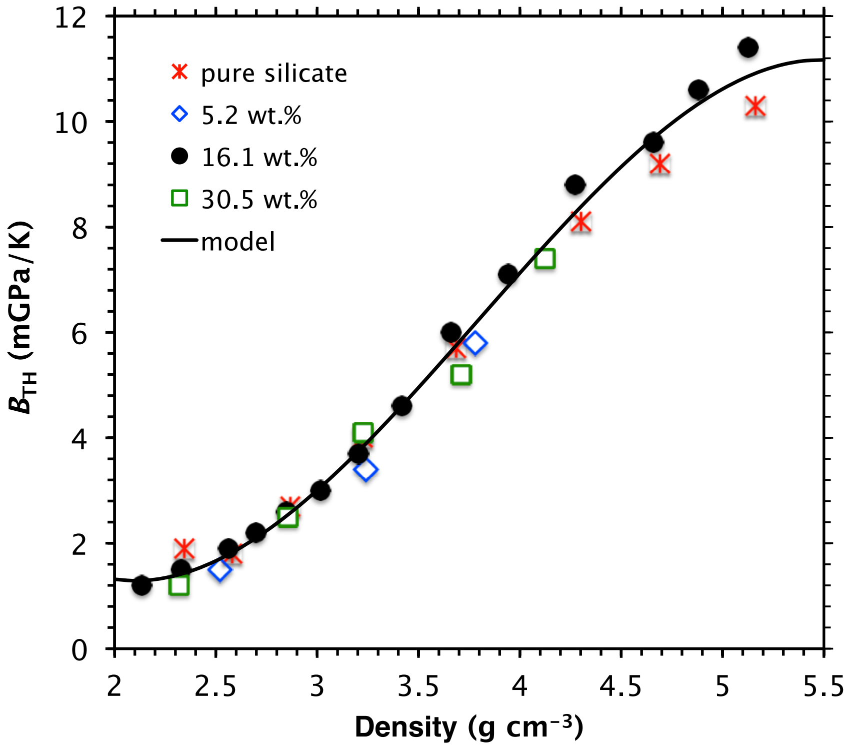

Supplementary figure S4. Density comparisons as a function of pressure at 4000 K between three cases of carbon incorporation as CO_2, CO and C in $MgSiO_3$ liquid for the carbon concentrations of 16.1 wt.% CO_2 and their equivalent CO and C amounts. Also shown is the density-pressure profile of the pure $MgSiO_3$ liquid.

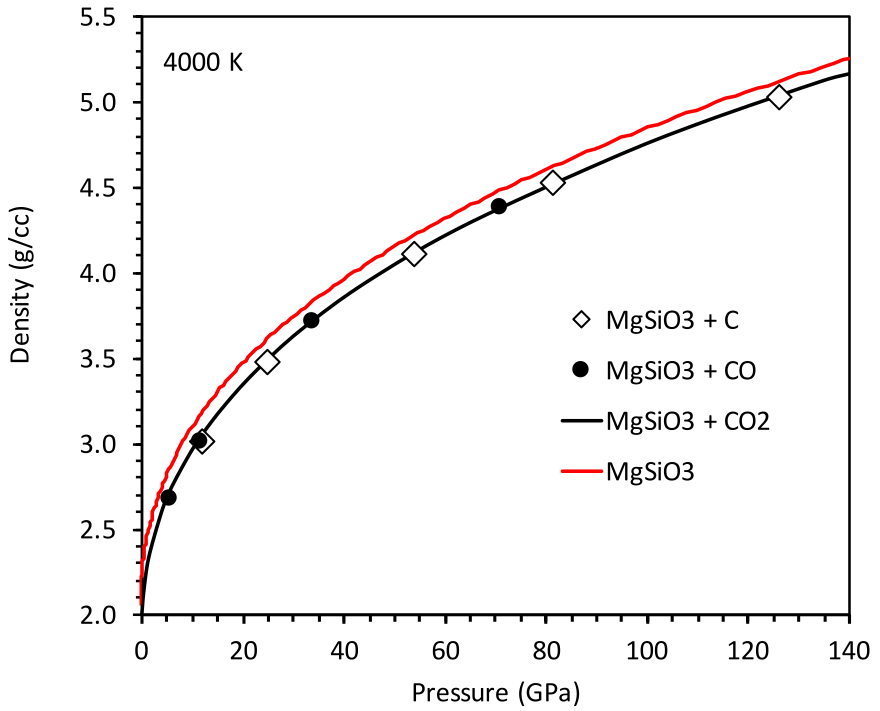

Supplementary figure S5. Mean square displacement (MSD) as a function of time for Mg, Si, O and C atoms for CO_2-bearing silicate melt at the following three conditions:

2200 K and 0 GPa (top)

3000 K and 18 GPa (middle)

4000 K and 92 GPa (bottom)

As can be seen, all MSD curves show three regimes. In the initial balistic regime (below 0.01 ps), MSD is quadratic in time. The middle regime extending up to 1 ps represents the cage effect where the atom gets trapped in a local neighbourhood. Finally, beyond 1 ps in the diffusive regime, the MSD varies linearly with time as shown by the unity slope in the log-log plot. Thus, a diffusive regime has been reached for the simulated liquid system at each condition for all atomic species. Also, MSD exceeds at least 20 Å^2, which implies that even the slowest species (Si) has moved 4.5 Å on average, i.e., more than a couple of Si-O bond lengths.

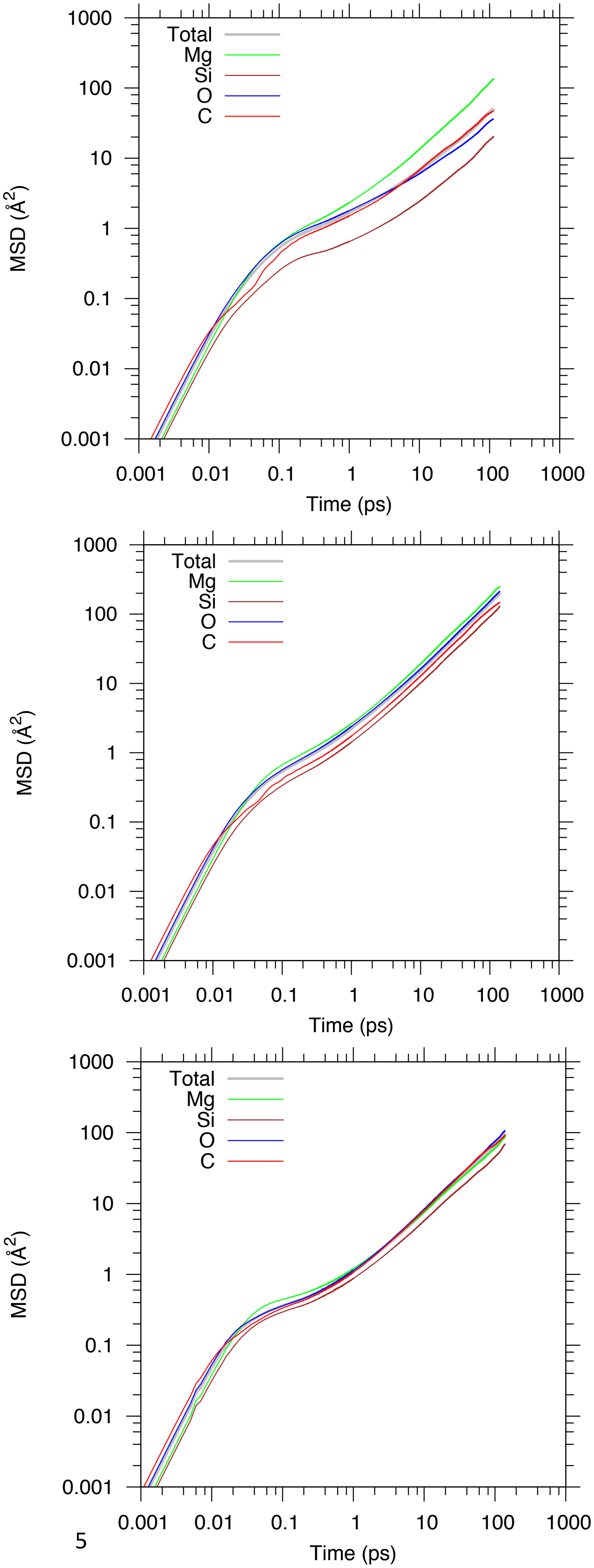

Supplementart text 1. Estimation of density of $MgSiO_3$ liquid by including iron.

The iron partitioning coefficient between the solid (S) and liquid (L) phases of $(Mg,Fe)SiO_3$ system is expressed as:

$$K_{Fe} = (Fe_S/Fe_L)\ (1 - Fe_L)/(1 - Fe_S)$$

where Fe_S and Fe_L represent the fractions of iron contained in the solid and molten silicates, respectively. The measured data K_{Fe} for $(Mg,Fe)SiO_3$ system are 0.3–0.4 at 25 GPa (Corgne, et al. 2005) , dropping below 0.1 above 75 GPa (Nomura, et al. 2011). Measurements on Al-bearing silicates are controversial, reporting low values of around 0.2 (Tateno, 2014) as well relatively high values around 0.5 (Andrault, et al. 2012). The other relevant quantity is the bulk iron content of the mantle defined as $X_{Fe} = Fe / (Mg + Fe)$.

Consider a partially molten mantle with melt fraction of ϕ_m. The equation balancing the amounts of iron between the solid mantle and partial melt is

$$X_{Fe} = \phi_m\ Fe_L + (1\text{-}\phi_m)\ Fe_S$$

Generally, the amount of partial melt expected in the deep mantle is very small like 1%, i.e., $\phi_m = 0.01$. So, the above equation can be approximated by ignoring the term containing ϕ_m:

$$X_{Fe} = Fe_S$$

The bulk iron content of the mantle is often taken to be $X_{Fe} = 0.1$, so we have $Fe_S = 0.1$. The partition coefficient equation can be also approximated as

$$K_{Fe} = (0.1/Fe_L)\ (1 - Fe_L)/(1 - 0.1) = 0.11\ (1\text{-}Fe_L)/Fe_L$$

Solving for Fe_L, we have

$$Fe_L = 0.11 / (K_{Fe}+0.11)$$

Consider the following two cases of iron partitioning between the solid mantle and partial melt.

First, at 23.6 GPa corresponding to 660 km depth, we take $K_{Fe} = 0.4$ so we have $Fe_L = 0.11/(0.4+0.11) = 0.22$. We then estimate the melt density of $(Mg_{0.78},Fe_{0.22})SiO_3$ with 5 wt.% CO_2 along the 2000 K isotherm for comparing with the mantle density at the 660 km depth.

Second, at 135 GPa corresponding to the core-mantle boundary, we take $K_{Fe} = 0.3$ so we have $Fe_L = 0.11/(0.3+0.11) = 0.27$. We then estimate the melt density of $(Mg_{0.73},Fe_{0.27})SiO_3$ with 5 wt.% CO_2 along the 4000 K isotherm for comparing the mantle density at the CMB.

References

1. Andrault, D. et al. Solid-liquid iron partitioning in Earth's deep mantle. *Nature* **487**, 354-357 (2012).
2. Corgne, A., Liebske, C., Wood, B. J., Rubie, D. C. & Frost, D. J. Silicate perovskite-melt partitioning of trace elements and geochemical signature of a deep perovskitic reservoir. *Geochim. Cosmochim. Acta* **69**, 485–496 (2005).
3. Nomura, R. et al. Spin crossover and iron-rich silicate melt in the Earth's deep mantle. *Nature* **473**, 199-202 (2011).
4. Tateno, S., Hirose, K. & Ohishi, Y. Melting experiments on peridotite to lowermost mantle conditions. *J. Geophys. Res. Solid Earth* **119**, 4684-4694 (2014).
